# Enhancing Post-Mastectomy Care: Telehealth’s Impact on Breast Reconstruction Accessibility for Breast Cancer Patients

**DOI:** 10.3390/cancers16142555

**Published:** 2024-07-16

**Authors:** Stephen A. Stearns, Daniela Lee, Valeria P. Bustos, Anthony Haddad, Natalie Hassell, Erin Kim, Jose A. Foppiani, Theodore C. Lee, Samuel J. Lin, Bernard T. Lee

**Affiliations:** Beth Israel Deaconess Medical Center, Division of Plastic and Reconstructive Surgery, Harvard Medical School, Boston, MA 02215, USA

**Keywords:** telemedicine, breast reconstruction, breast cancer care

## Abstract

**Simple Summary:**

This research explores how the increase in telemedicine during the COVID-19 pandemic has impacted the accessibility of breast reconstruction for breast cancer patients. This study compares patient data from before and during the widespread adoption of telehealth, focusing on how far patients traveled for surgery and their follow-up care. The findings indicate that while the rate of breast reconstruction surgeries remained consistent, telehealth significantly enhanced follow-up care, suggesting it plays a crucial role in improving healthcare access and continuity for breast cancer patients. This underscores the potential of telemedicine to bridge gaps in healthcare delivery, particularly for post-mastectomy care.

**Abstract:**

Objective: To examine how the recent sharp rise in telemedicine has impacted trends in accessibility of breast reconstruction (BR). Patients and Methods: A retrospective study reviewed patients who underwent a total mastectomy at our institution from 1 August 2016 to 31 January 2022. By comparing cohorts before and during the widespread implementation of telemedicine, we assessed telehealth’s impact on healthcare accessibility, measured by distance from patients’ residences to our institution. Results: A total of 359 patients were included in this study. Of those, 176 received total mastectomy prior to the availability of telemedicine, and 183 in the subsequent period. There were similar baseline characteristics among patients undergoing mastectomy, including distance from place of residence to hospital (*p* = 0.67). The same proportion elected to receive BR between groups (*p* = 0.22). Those declining BR traveled similar distances as those electing the procedure, both before the era of widespread telemedicine adoption (40.3 and 35.6 miles, *p* = 0.56) and during the height of telemedicine use (22.3 and 61.3 miles, *p* = 0.26). When tracking follow-up care, significantly more patients during the pandemic pursued at least one follow-up visit with their original surgical team, indicative of the increased utilization of telehealth services. Conclusions: While the rate of BR remained unchanged during the pandemic, our findings reveal significant shifts in healthcare utilization, highly attributed to the surge in telehealth adoption. This suggests a transformative impact on breast cancer care, emphasizing the need for continued exploration of telemedicine’s role in enhancing accessibility and patient follow-up in the post-pandemic era.

## 1. Introduction

Breast cancer is a devastating disease with more than 280,000 new diagnoses and nearly 50,000 resulting deaths each year. Approximately 12.9% of women will be diagnosed with breast cancer in their lifetime, making it the second most common type of cancer in women behind melanoma [1]. Advances in screening and adjuvant therapies have dramatically improved outlooks for this disease. Models of breast cancer trends estimate a 49% reduction in mortality due to advancements in mammography and medical treatment [2]. The development of comprehensive screening protocols and technology paired with effective adjuvant chemotherapy and hormonal treatments have resulted in a 90.3% 5-year survival rate and as high as a 99.0% 5-year survival when initially found as confined to a primary site [1].

A crucial component to the continued improvement of breast cancer treatment is ensuring widespread access to affordable and comprehensive care. In 1998, U.S. legislation mandated insurance coverage of breast reconstruction after mastectomy, recognizing the importance of eliminating financial barriers for reconstruction [3]. Despite the subsequently increased reconstruction rates, disparities in utilization of services continues [4]. Age and race independently impact breast reconstructive choices, with younger, white women pursuing reconstructive surgery at significantly higher rates than their older and racial minority counterparts [5]. Rural versus urban settings, private versus academic centers, and level of patient education were all associated with varying breast reconstruction rates, suggesting that the lack of access to breast reconstructive services is key to its underutilization [6].

In recent years, telemedicine has emerged as a potential solution to address disparities in access to healthcare services, including breast reconstruction. Telemedicine, or telehealth, utilizes telecommunication technology, such as computers and smartphones, to enable remote consultations between patients and healthcare providers. This resource offers a convenient and accessible alternative to traditional in-person visits. The recent COVID-19 pandemic accelerated the adoption of telemedicine, as healthcare systems sought to maintain continuity while minimizing the risk of viral transmission [7]. The implementation of telemedicine can partly be attributed to the expansion of insurance coverages. Many insurance companies added temporary approval of telehealth services, which led to increased utilization [8]. For example, Medicare patients were offered the same telemedicine coverage as the Medicare ‘Advantage’ patients. There was also a rise in state and federal government funding to promote telehealth in low-income areas in addition to providing necessary technology to complete remote visits [9]. One of the biggest pushes for telehealth integration was when the Office of Civil Rights, of the U.S. Department of Health and Human Services, announced that potential HIPAA violations for using everyday communication technology for patient care would be waived [10]. While the impact of telemedicine on breast reconstruction has yet to be fully explored, there is growing evidence that it may play a significant role in improving access to these services for underserved populations as these individuals have a greater willingness to use this technology [11].

Physical distance from a surgical center can be used as a proxy to assess access to breast reconstruction. One study finds that women undergoing breast reconstruction travel significantly farther distances to reach their surgical site than those opting for mastectomy alone [12]. This trend is not limited to the United States, with similarly reported trends present in Canada, where the financial burden of medical care is significantly different, yet the barrier of distance to care persists [13]. The concept of physical distance impairing access to breast reconstructive services is well-established in the literature, yet the effect of the sharp rise in telehealth in the past few years on this trend has yet to be investigated. This study is the first of its kind to explore how disparities in access to breast reconstructive services have changed due to the recent paradigm shift in healthcare delivery.

## 2. Methods

### 2.1. Study Setting and Design

A retrospective observational study was performed, with adherence to STROBE guidelines, on patients who underwent total mastectomy with or without breast reconstruction at a single institution from August 2016 to January 2022 [14]. A query was conducted through our institution’s I2B2 system (Clinical Query 2 software (Version 1.0.2)) to gather information from our Clinical Data Repository. Patients’ demographics and clinical characteristics were extracted and recorded using REDCap (Research Electronic Data Capture) electronic data capture tools hosted at our institution [15,16]. The cohort was divided into two: (1) the pre-telemedicine group (i.e., those receiving mastectomy in the pre-pandemic, before 2020) and (2) the telemedicine group (i.e., those undergoing mastectomy during the pandemic, starting in 2020).

### 2.2. Variables

Demographic characteristics were retrieved from the retrospective chart review, including the following: age, sex, language, marital status, race, ethnicity, and education. Each patient’s home address was collected to identify the distance between residence and hospital. The length from the patient’s address to our institution was calculated using an online geodesic distance calculator (Measure Distances, Chris Bell, London, UK). This measurement was used as a surrogate variable for healthcare access. Additionally, clinical characteristics of type of breast reconstruction and follow-up time were extracted. Follow-up time was measured from the surgery date to the most recent follow-up visit with the operating team (either breast or plastic and reconstructive clinic).

### 2.3. Aims

The aims of this study were threefold. First, we sought to assess differences in the distance patients traveled to access breast reconstructive services before and during the recent telemedicine surge. Our second aim was to evaluate differences in breast reconstruction rates between cohorts. Lastly, we aimed to identify differences in post-operative follow-up with the original surgical team.

### 2.4. Statistical Analysis

For descriptive statistics, means and standard deviations were used for continuous variables, and frequencies and percentages were used for categorical variables. For inferential analysis, measures of distance between residence and hospital, follow-up, and breast reconstructive decisions were used to supplement the analysis. Unpaired Student’s *t* and chi-square tests were performed to assess differences between groups. Statistical significance was set up for a *p*-value less than 0.05 except in post hoc chi-square tests, where the Bonferroni Adjustment was used to set the statistical significance to counteract Type I error. Statistical analysis was conducted in JMP Pro 17 statistical software (JMP^®^, Version 17.0.0. SAS Institute Inc., Cary, NC, USA, 1989–2023).

## 3. Results

### 3.1. Demographics

A total of 359 patients were included in our analysis. Of those, 176 (49.0%) and 183 (51.0%) patients corresponded to the pre-telemedicine and telemedicine groups, respectively. The mean (SD) age of patients in the overall cohort was 55.5 (13.9). The pre-telemedicine’s group mean age was 56.4 (15.3), and the telemedicine group’s mean age was 54.6 (12.4). Everyone in the sample was female, and most were English-preferring. Among patients in the study, 249 (69.4%) identified as White, followed by Asian in 42 cases (11.7%). The cohort mainly identified as non-Hispanic. Both groups had similar baseline characteristics. A summary of the patients’ demographics divided by telemedicine adoption period is depicted in Table 1.

### 3.2. Distance and Breast Reconstruction Decision

**Figure 1:**
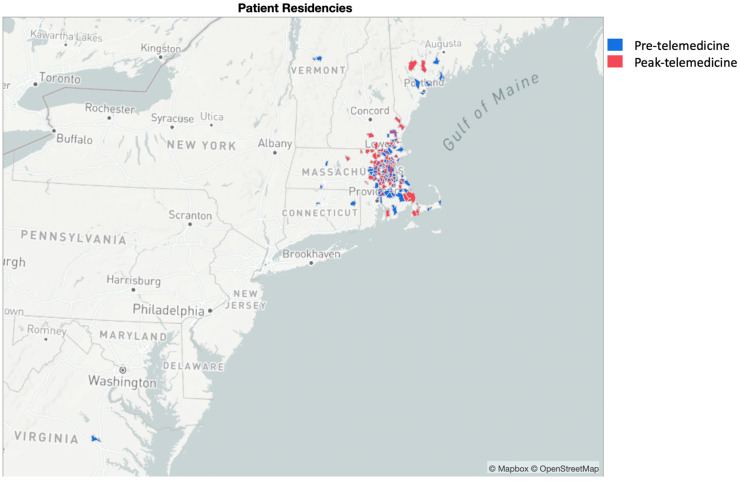
Map of pre-telemedicine and peak-telemedicine cohort residencies. Figure uses map data from © Mapbox and © OpenStreetMap and their data sources, which are available under the Open Database License. To learn more, visit https://www.mapbox.com/about/maps/ (accessed on 5 July 2024) and http://www.openstreetmap.org/copyright (accessed on 5 July 2024).

When comparing the distance from the place of residence to the hospital, there was no significant difference found between the pre-telemedicine era and the telemedicine era (25.7 and 15.1 miles, respectively, *p* = 0.67). In our cohort, before the rise of telehealth, those declining to undergo breast reconstruction traveled equally far as those electing to do so (40.3 miles versus 35.6 miles, *p* = 0.56). This difference increased afterwards but was not statistically significant (22.3 miles versus 61.3 miles, *p* = 0.26). Further examining patients undergoing reconstruction, there was no difference in distance traveled for autologous versus implant-based breast reconstructions (pre-telemedicine *p* = 0.47, telemedicine *p* = 0.19) (Figure 1 and Table 2).

There was a significant increase in use of telemedicine for the first breast surgery or plastic surgery consultation, confirming our method of grouping (pre-telemedicine: 1.7%, telemedicine: 29.0%, *p* < 0.001). A post hoc z-test on the adjusted residuals with Bonferroni correction revealed that only for those who specified “Yes” or “No” was there a significant difference between the pre-telemedicine and peak-telemedicine time periods (*p* < 0.008). Additionally, when tracking follow-up care, more patients during the telemedicine period pursued at least one follow-up visit with their original surgical team (pre-telemedicine: 67.6%, telemedicine: 84.7%, *p* < 0.001). From all the cohorts, 85 (23.7%) patients did not pursue any type of follow-up visit with the original surgical team and were deemed “lost to follow-up”. The mean time from procedure to most recent follow-up visits for those not lost to follow-up was greater in the pre-telemedicine cohort at 566.5 days compared to the telemedicine cohort at 286.8 days (*p* < 0.001) (Table 2).

### 3.3. Clinical Characteristics

Overall, 208 (58.0%) patients decided to undergo breast reconstruction after mastectomy. Comparing the pre-telemedicine and peak-telemedicine groups, a similar proportion of patients elected to receive breast reconstruction (54.5% and 61.2%, *p* = 0.22) (Table 3). Most patients in both cohorts underwent immediate tissue expander placement with delayed reconstruction. Of those that completed their breast reconstruction, 113 (68.5%) elected for implant-based breast reconstruction and 52 (31.5%) chose autologous breast reconstruction. The choice of implant versus autologous implant varied significantly between cohorts, with 64 (82.1%) receiving implant-based reconstruction and 14 (18.0%) receiving autologous reconstruction pre-telemedicine, versus 49 (56.3%) and 38 (43.7%) in the peak-telemedicine cohort, respectively (*p* < 0.001) (Table 4).

## 4. Discussion

The global healthcare landscape has undergone significant changes in recent times, marked by a paradigm shift towards the accelerated adoption of telemedicine. This transformative period has brought about notable adjustments in breast cancer care, with health systems adapting to ensure continuity despite the recent pandemic and subsequent massive elective surgery cancellations [17]. During this time, telemedicine has emerged as a crucial tool for maintaining continuity of care and improving access to critical services. Due to the elective nature of breast reconstruction, patients with breast cancer likely experience frequent delays in receiving their care, further complicating their path to recovery [18,19]. Our study provides preliminary evidence that breast reconstructive services seem to have adapted adequately to the new digital era of health care access. Patients undergoing breast reconstruction were not found to travel farther for care than those who do not pursue reconstruction, regardless of the time period. Rates of reconstruction were also similar. The largest difference found in the telemedicine cohort was in behaviors of surgical follow-up.

Previous studies have demonstrated the impact of geographic barriers on breast reconstruction. Interestingly, this study’s pre-telemedicine and subsequent cohort findings contrast with existing data that demonstrate patients who undergo breast reconstruction travel farther to access services than those who do not [12]. Neither group of our study demonstrated differences in distance travelled to the hospital among post-mastectomy patients. A likely explanation for this discrepancy is that Boston, the location of our institution, is a medically saturated environment with multiple hospitals offering breast surgical care, and so utilization patterns understandably differ from the generalized national findings [12]. Given the number of reconstruction options available in the Boston market, distance may present less of a barrier in accessing this type of care. The consistent trend of equal travel distances, regardless of the pursuit of reconstruction, persisted during the prime era of telemedicine adoption.

Further stratifying distance traveled by type of reconstruction, Albornoz et al. found that patients who underwent autologous breast reconstruction had to travel farther than those who underwent implant-based breast reconstruction [12]. Our study’s findings showed that, despite a change in type of reconstruction received, there was no significant difference in distance traveled between types of reconstruction in either the pre-telemedicine or telemedicine cohorts. There are multiple possible explanations for this finding. Again, in a highly saturated medical market with ample medical resources available, multiple reconstructive options for patients mitigate distance as a barrier for selecting one type of reconstruction over another. Although the pandemic disrupted autologous breast reconstruction, our institution continued to offer both modalities to patients, and so, understandably, the rise of telehealth did not disproportionately affect one over the other.

Surprisingly, there did not appear to be a change in the rate of breast reconstruction. In the setting of record low visits to emergency departments and ongoing delayed health screenings, recent global events such as the pandemic have created a medical hesitancy in many areas of healthcare [20,21,22]. In breast reconstruction, however, the proportion of women with breast cancer electing to pursue reconstruction procedures did not change, reflecting the continued desire of many patients to receive this form of cancer care despite potential barriers. There was also a significant increase in autologous over implant-based reconstruction in the telemedicine cohort, possibly allowed by increased availability of recovery time at home and remote check-ins with providers. These findings have reassuring implications for breast reconstructive centers as it highlights the value of breast reconstruction in cancer treatment among women.

The final component of this study is the demonstrated increased rate of follow-up in the telemedicine cohort with the patient’s original surgical team. This change is likely positive, as continuity of care with a single medical team has consistently proven to result in superior outcomes [23,24]. As patients present for follow-up care to a familiar team, information fidelity surrounding a patient’s history and care decisions is maintained. This increased follow-up ensures improved detection of adverse outcomes and early intervention to correct any improper recovery. While the reason for this improvement in continuity is likely multifactorial, technological advancements paired with a shift in public perception of health both are likely contributory. There was a significant increase in use of telemedicine for breast surgery and plastic surgery consultations in the cohort of patients for 2020 and beyond. This trend suggests that patients are increasingly embracing this technology as a means of maintaining their care.

Increased ease of access to medical expertise through technological developments such as telemedicine allows patients to connect with their medical team without the need for travel or time commitment. Tan et al. report a nearly 50% reduction in no-show appointments using online health measures, with maintained quality of outcomes [25]. Through widespread adoption of this technology, the barriers of physical distance to access healthcare for a follow-up have decreased significantly. Technology also supported an increased ability to work from home, likely enabling patients more flexibility and ease in scheduling and attending follow-up visits. Studies have also shown that telemedicine can provide comparable quality of care to in-person consultations, particularly in the context of follow-up visits [26,27,28]. Additionally, this change in increased follow-up may derive from an altered perspective of health at a population level, potentially mirroring an increasingly health-conscious society. Recent studies demonstrate increased levels of health anxiety and corresponding improvement of certain health habits [29,30,31]. Regardless of the cause of the increased continuity of care, the shift towards improved follow-up is a promising finding.

**Figure 2:**
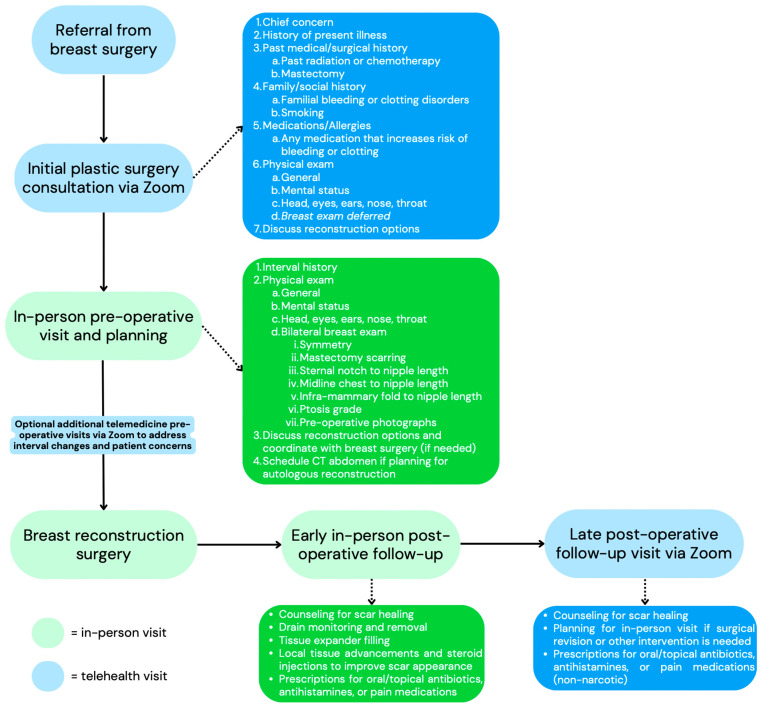
Flowchart of breast reconstruction patients’ care at our institution with detailed descriptions of services associated with the specific visit. Blue boxes represent appointments that can be converted to a telehealth format.

Beyond post-surgery follow-up visits, there is potential to expand telehealth use for other services that are needed for breast cancer patients. The National Coalition for Cancer Survivorship Telehealth Project demonstrated that the telehealth setting was most acceptable for second/third opinions, mental health visits, treatment monitoring, and post-operative or high-risk patient check-ins [32]. The virtual setting was least acceptable for first visits with a new physician, treatment planning, and patients with new or evolving symptoms [32]. These principles can also be applied in the setting of oncologic reconstruction. The teleconsultation model for breast reconstruction patients developed by Xue et. al. starts with getting a referral from a breast surgeon’s office, then having an initial telemedicine consultation with a plastic surgeon on Zoom that occurs in six sequential steps incorporating nurse intake, markings, and a visual physical exam [33]. This visit is followed by a multidisciplinary in-person meeting with all involved specialties, including breast surgery, plastic surgery, and anesthesia, with a post-operative follow-up via telemedicine [33]. Our practice follows a similar model, with a low threshold to initiate future in-person visits based on patient presentation, concerns, and symptoms (Figure 2).

Our findings also prompt an exploration into the role of insurance reimbursement for telehealth, and how this influences patient access to reconstructive care. In the past, telemedicine was not always covered by insurance. However, many insurance companies now cover these services for breast reconstruction care, either at the same rate as in-person services or at a reduced rate. The U.S. Department of Health and Human Services took a range of administrative steps to expedite the adoption and awareness of telehealth during the recent public health emergency. Some of these telehealth policy changes were made permanent, such as making rural emergency hospitals eligible originating sites for telehealth. Many substantial temporary changes were made through 31 December 2024, including the following: Federally Qualified Health Centers and Rural Health Clinics can serve as a distance site provider for telehealth services, Medicare patients can receive telehealth services in their home, geographic restrictions were completely lifted, and distant telehealth services were allowed to be provided by all eligible Medicare providers [34]. These shifts in insurance coverage have made it more affordable for patients to access telemedicine services, and it has also made it more likely that healthcare providers will offer these services.

This study is not without its limitations. Specifically, it describes altered follow-up practices with the original surgical team; however, due to the nature of a single-institutional study, we did not assess the concept of follow-up in its entirety, as it is unknown whether patients pursued follow-up care at an outside hospital. This limits some of the claims that can be made regarding this variable. Additionally, the relationship between increased follow-up and the rise of telemedicine is an associative one, and thus we cannot assert a causal connection between variables. Instead, the fact that a statistical relationship exists provides initial evidence that there may be some connection between variables and indicates an area for further study. Finally, the data may have been too macroscopic, not adequately representing the vastly different healthcare landscape during the pandemic from month to month as systems adapted. Grouping all patients into a single group may have blurred some of the changes brought on by other external factors.

As we move forward, it is evident that telemedicine will play an increasingly central role in the delivery of breast reconstruction care. Healthcare providers and institutions should continue to invest in developing and expanding telemedicine infrastructure and training to ensure that patients have equitable access to these services. Furthermore, future research efforts should focus on evaluating the long-term impact of telemedicine on breast reconstruction outcomes, identifying potential areas for improvement, and optimizing telemedicine protocols to maximize patient satisfaction and clinical effectiveness.

## 5. Conclusions

Despite the consistent rate of breast reconstruction procedures during the period of widespread telemedicine adoption, notable shifts in healthcare utilization were observed in the subsequent cohort, suggesting an association with the integration of telehealth services. Notably, patients in the post-telemedicine adoption era demonstrated an increased inclination towards seeking post-operative follow-up with their original surgical team. Remarkably, the previously reported barrier of distance, known to disproportionately affect patients undergoing breast reconstruction, did not manifest as a significant factor before or during the era of telemedicine adoption. While the comprehensive impact of the paradigm shift towards telemedicine remains to be fully understood, our findings underscore its transformative influence on patient decision-making in the realm of breast cancer care.

## Figures and Tables

**Table 1:** Patient demographics.

	Pre-Telemedicine (*n* = 176)	Peak-Telemedicine (*n* = 183)	*p* Value
Age *mean* (SD ^a^)	56.4 (15.3)	54.6 (12.4)	0.21
Sex *n* (%)			
Female	176 (100%)	183 (100%)	
Marital status *n* (%)			
Single	8 (4.5%)	5 (2.7%)	0.92
Married	50 (28.4%)	43 (23.5%)	
Divorced	2 (1.1%)	2 (1.1%)	
Widowed	4 (2.3%)	2 (1.1%)	
Not specified	112 (63.6%)	131 (71.6%)	
Ethnicity *n* (%)			
Hispanic	8 (4.5%)	19 (10.4%)	0.09
Non-Hispanic	150 (85.2%)	150 (82.0%)	
Not specified	18 (10.2%)	14 (7.7%)	
Race *n* (%)			
White	119 (67.6%)	130 (71.0%)	0.81
American Indian	1 (0.6%)	0 (0%)	
Asian	21 (11.9%)	21 (11.5%)	
Black	19 (10.8%)	20 (10.9%)	
Not specified	16 (9.1%)	12 (6.6%)	
Language *n* (%)			
English	78 (44.3%)	58 (31.7%)	0.68
Spanish	2 (1.1%)	4 (2.2%)	
Japanese	1 (0.6%)	0 (0%)	
Mandarin	1 (0.6%)	0 (0%)	
Cantonese	4 (2.3%)	3 (1.6%)	
Vietnamese	0 (0%)	1 (0.5%)	
Not specified	90 (51.1%)	117 (63.9%)	
Education *n* (%)			
Less than college degree	53 (30.1%)	68 (37.2%)	0.13
College or higher	101 (57.4%)	102 (55.7%)	
Not specified	22 (12.5%)	13 (7.1%)	

^a^ SD = standard deviation.

**Table 2:** Healthcare access.

	Pre-Telemedicine (*n* = 176)	Peak-Telemedicine (*n* = 183)	*p* Value
Distance in miles *mean* (SD ^a^)	36.7 (71.3)	45.9 (282.3)	0.67
Telemedicine ^1^ *n* (%)			
Yes	3 (1.7%)	53 (29.0%)	<0.001
No	148 (84.1%)	115 (62.8%)	
Not specified	25 (14.2%)	15 (8.2%)	
Follow-up ^2^ *n* (%)			
Yes	119 (67.6%)	155 (84.7%)	<0.001
No	57 (32.4%)	28 (15.3%)	
Days of follow-up *mean* (SD ^a^)	566.5 (427.5)	286.8 (168.9)	<0.001

^1^ Refers to telemedicine use for first breast surgery or plastic surgery consultation. ^2^ Refers to either in-person or virtual follow-up visits post-operatively. ^a^ SD = standard deviation.

**Table 3:** Clinical characteristics.

	Pre-Telemedicine (*n* = 176)	Peak-Telemedicine (*n* = 183)	*p* Value
Mastectomy indication *n* (%)			
Prophylactic	14 (8.0%)	15 (8.2%)	0.83
Unilateral breast cancer	94 (53.4%)	90 (49.2%)	
Bilateral breast cancer	11 (6.3%)	15 (8.2%)	
Unilateral cancer with prophylactic surgery of the other breast	57 (32.4%)	63 (34.3%)	
Breast reconstruction *n* (%)			
Yes	96 (54.5%)	112 (61.2%)	0.22
No	80 (45.5%)	71 (38.8%)	

**Table 4:** Breast reconstruction characteristics.

	Pre-Telemedicine (*n* = 96)	Peak-Telemedicine (*n* = 112)	*p* Value
Timing *n* (%)			
Immediate reconstruction	15 (15.6%)	15 (13.4%)	0.88
Delayed reconstruction	10 (10.4%)	13 (11.6%)	
Immediate TE with delayed reconstruction	71 (74.0%)	84 (75.0%)	
Completion *n* (%)			
Yes	78 (81.3%)	87 (78.4%)	0.61
No	18 (18.8%)	24 (21.6%)	
Type ^a^ *n* (%)			
Implant	64 (82.1%)	49 (56.3%)	<0.001
Autologous	14 (18.0%)	38 (43.7%)	
DIEP	11 (78.6%)	33 (84.6%)	0.40
TRAM	1 (7.1%)	0 (0.0%)	
Latissimus dorsi	2 (14.3%)	6 (15.4%)	

^a^ Out of the patients who completed breast reconstruction.

## Data Availability

The data presented in this study are available on request from the corresponding author. The data are not publicly available due to privacy restrictions.
